# Supplementary material for: Asymmetric activity of NetrinB controls laterality of the Drosophila brain
Source: Nat Commun. 2023 Feb 24;14:1052. doi: 10.1038/s41467-023-36644-4 (PMC9958012; doi:10.1038/s41467-023-36644-4)
Supplement: Supplementary file 1 — Supplementary Information [file 41467_2023_36644_MOESM1_ESM.pdf]

# Asymmetric activity of NetrinB controls laterality of the *Drosophila* brain

## Supplementary Information

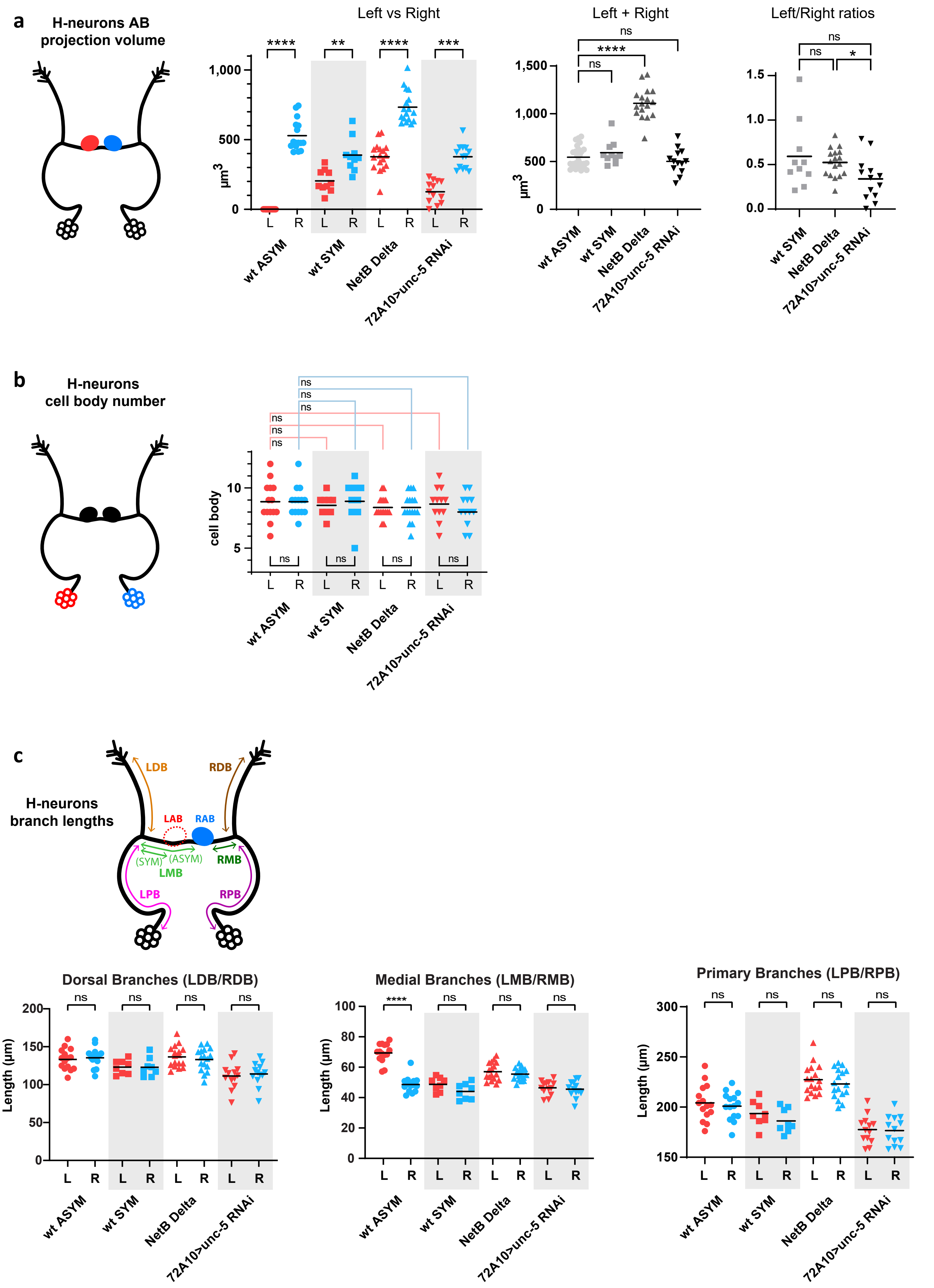

a
H-neurons AB projection volume
Left vs Right
µm3
1,000
500
0
****
**
****
***
L
R
wt ASYM
wt SYM
NetB Delta
72A10>unc-5 RNAi
Left + Right
ns
****
ns
1,500
1,000
500
0
Left/Right ratios
ns
ns
*
1.5
1.0
0.5
0.0
b
H-neurons cell body number
cell body
10
5
ns
c
H-neurons branch lengths
LDB
RDB
LAB
RAB
(SYM)
(ASYM)
LMB
RMB
LPB
RPB
Dorsal Branches (LDB/RDB)
Length (µm)
200
150
100
50
0
Medial Branches (LMB/RMB)
100
80
60
40
20
0
Primary Branches (LPB/RPB)
300
250
200
150

## Supplementary Figure 1: Morphometric analysis of adult asymmetric H-neurons in wild-type, and *netB/unc-5* loss-of -unction conditions

**a**. Comparison of H-neurons AB projection volume in wild-type (ASYM, SYM), *netB* mutant and “72A10-Gal4>UAS-unc-5 RNAi” flies indicates that all “SYM” conditions retain significant asymmetric bias with the left projections occupying a smaller volume as compared to the right projections (ASYM flies, left and right: n=16; SYM flies, left and right: n=10; NetB mutant flies, left and right: n=17; 72A10-Gal4>UAS-unc-5 RNAi flies, left and right: n=13; n numbers correspond to the numbers of hemispheres analysed). Total volume of Left and Right AB projections is similar in wild-type ASYM, wild-type SYM and “72A10-Gal4>UAS-unc-5 RNAi” flies (ASYM flies: n=16, SYM flies: n=10; NetB mutant flies: n=17; 72A10-Gal4>UAS-unc-5 RNAi flies: n=13; n numbers correspond to the numbers of brains analysed). While *netB* mutant and wild-type SYM flies have comparable Left/Right AB projection volume ratio, this ratio is significantly smaller in “72A10-Gal4>UAS-unc-5 RNAi” flies as compared to *netB* mutant flies (SYM flies: n=10; NetB mutant flies: n=17; 72A10-Gal4>UAS-unc-5 RNAi flies: n=13; n numbers correspond to the numbers of brains analysed). P-values are calculated with two-tailed Wilcoxon test. P-values are: AB volume Left vs Right: wt ASYM: <0.0001; wt SYM: 0.0098; NetB Delta: <0.0001; 72A10>unc-5 RNAi: 0.0002; AB Volume Left+Right wt ASYM vs NetB Delta: <0.0001. Source data are provided as a Source Data file.

**b**. The number of cell bodies do not show LR asymmetry in any of the conditions (ASYM flies, left and right: n=14; SYM flies, left and right: n=9; NetB mutant flies, left and right: n=16 ; 72A10-Gal4>UAS-unc-5 RNAi flies, left and right: n=12; n numbers correspond to the numbers of hemispheres analysed). P-values are calculated with two-tailed Wilcoxon test. Source data are provided as a Source Data file.

**c**. Length of primary and dorsal H neuron branches do not show asymmetry in wild-type, NetB mutant or 72A10-Gal4, UAS-unc-5 RNAi flies. Length of medial H neuron branches is also symmetric in wild-type SYM, NetB mutant or 72A10-Gal4, UAS-unc-5 RNAi flies (ASYM flies, left and right: n=15 ; SYM flies, left and right: n=8 ; NetB mutant flies, left and right: n=17 ; 72A10-Gal4>UAS-unc-5 RNAi flies, left and right: n=13; n numbers correspond to the numbers of hemispheres analysed). P-values are calculated with two-tailed Wilcoxon test. P-values for wt ASYM medial branch lengths: <0.0001. Source data are provided as a Source Data file.

Horizontal bar in plots represent mean value. Significance threshold for p-Values are: ns, non-significant; *, <0.05 ; **, <0.01 ; ***, < 0.001 ; ****, < 0.0001. All calculated p-Values are listed in Supplementary Data 3.

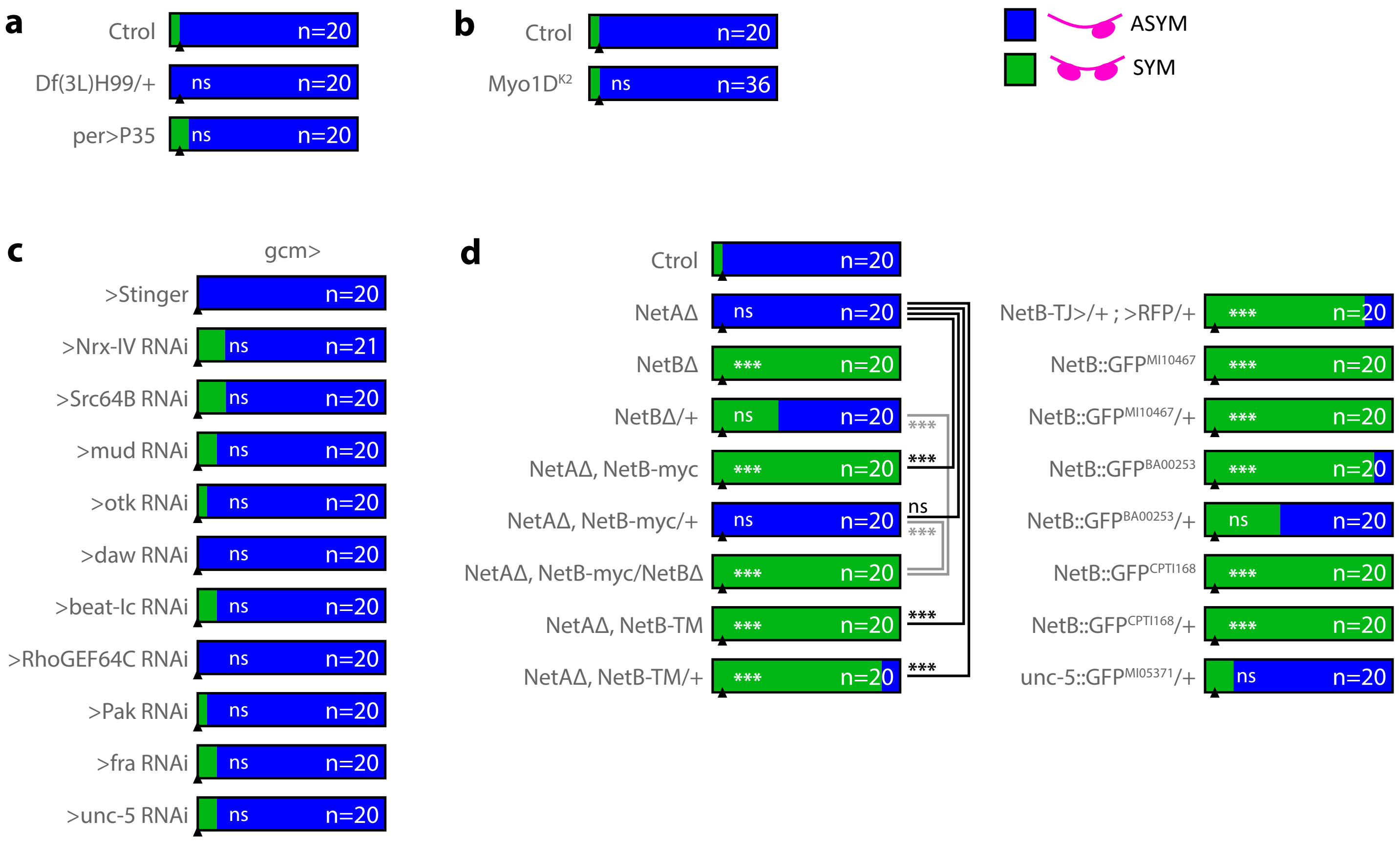
a
Ctrol
n=20
Df(3L)H99/+
ns
n=20
per>P35
ns
n=20
b
Ctrol
n=20
Myo1D^K2
ns
n=36
ASYM
SYM
c
gcm>
>Stinger
n=20
>Nrx-IV RNAi
ns
n=21
>Src64B RNAi
ns
n=20
>mud RNAi
ns
n=20
>otk RNAi
ns
n=20
>daw RNAi
ns
n=20
>beat-Ic RNAi
ns
n=20
>RhoGEF64C RNAi
ns
n=20
>Pak RNAi
ns
n=20
>fra RNAi
ns
n=20
>unc-5 RNAi
ns
n=20
d
Ctrol
n=20
NetAΔ
ns
n=20
NetBΔ
***
n=20
NetBΔ/+
ns
n=20
NetAΔ, NetB-myc
***
n=20
NetAΔ, NetB-myc/+
ns
n=20
NetAΔ, NetB-myc/NetBΔ
***
n=20
NetAΔ, NetB-TM
***
n=20
NetAΔ, NetB-TM/+
***
n=20
NetB-TJ>/+ ; >RFP/+
***
n=20
NetB::GFP^MI10467
***
n=20
NetB::GFP^MI10467/+
***
n=20
NetB::GFP^BA00253
***
n=20
NetB::GFP^BA00253/+
ns
n=20
NetB::GFP^CPTI168
***
n=20
NetB::GFP^CPTI168/+
***
n=20
unc-5::GFP^MI05371/+
ns
n=20

## Supplementary Figure 2: Additional phenotypic analysis

**a**. Blocking apoptosis using either the Df(3L)H99 allele (deletion of the pro-apoptotic genes *grm*, *hid* and *rpr*[65]) or by overexpressing the baculovirus caspase inhibitor P35 protein[66] using the per-Gal4 driver do not induce a H-neurons asymmetry phenotype. **b**. myo1D null mutant do not induce an H-neurons asymmetry phenotype. **c**. RNAi lines causing an asymmetry phenotype when expressed using the pan-neuronal driver, do not induce an asymmetry phenotype when expressed using the pan-glial driver gcm-Gal4. **d**. While the unc-5::GFP fusion protein do not cause an asymmetry phenotype, various NetB reporters (tagged, GFP fusion or Gal4 insertion) are non-functional alleles inducing H-neurons asymmetry phenotype similar to NetB null mutant phenotype. P-values are calculated with Pearson's Chi-squared test and Benjamini & Yekutieli multiple comparisons correction. P-values are: control vs NetB-Delta: 1.09E-07; control vs NetA-Delta. NetB-myc: 1.09E-07; control vs NetA-Delta. NetB-myc/NetB-Delta: 1.09E-07; control vs NetA-Delta. NetB-TM: 1.09E-07; control vs NetA-Delta. NetB-TM/+: 2.44E-06; control vs NetB-TJ-Gal4/+;UAS-RFP/+: 9.18E-06; control vs NetB::GFP MI10467: 1.09E-07; control vs NetB::GFP MI10467/+: 1.01E-05; control vs NetB::GFP BA00253: 5.15E-07; control vs NetB::Venus CPTI00168: 1.54E-07; control vs NetB::Venus CPTI00168/+: 1.09E-07; NetA-Delta vs NetA-Delta. NetB-myc: 5.15E-09; NetA-Delta vs NetA-Delta. NetB-TM: 5.15E-09; NetA-Delta vs NetA-Delta. NetB-TM/+: 1.20E-07; NetA-Delta. NetB-myc/NetB-Delta vs NetA-Delta. NetB-myc/+: 5.62E-09; NetA-Delta. NetB-myc/NetB-Delta vs NetB-Delta/+: 7.65E-05. Significance threshold for p-Values are: ns, non-significant; *, <0.05 ; **, <0.01 ; ***, < 0.001. All calculated p-Values are listed in Supplementary Data 3. n numbers correspond to the numbers of brains analysed. Source data are provided as a Source Data file.

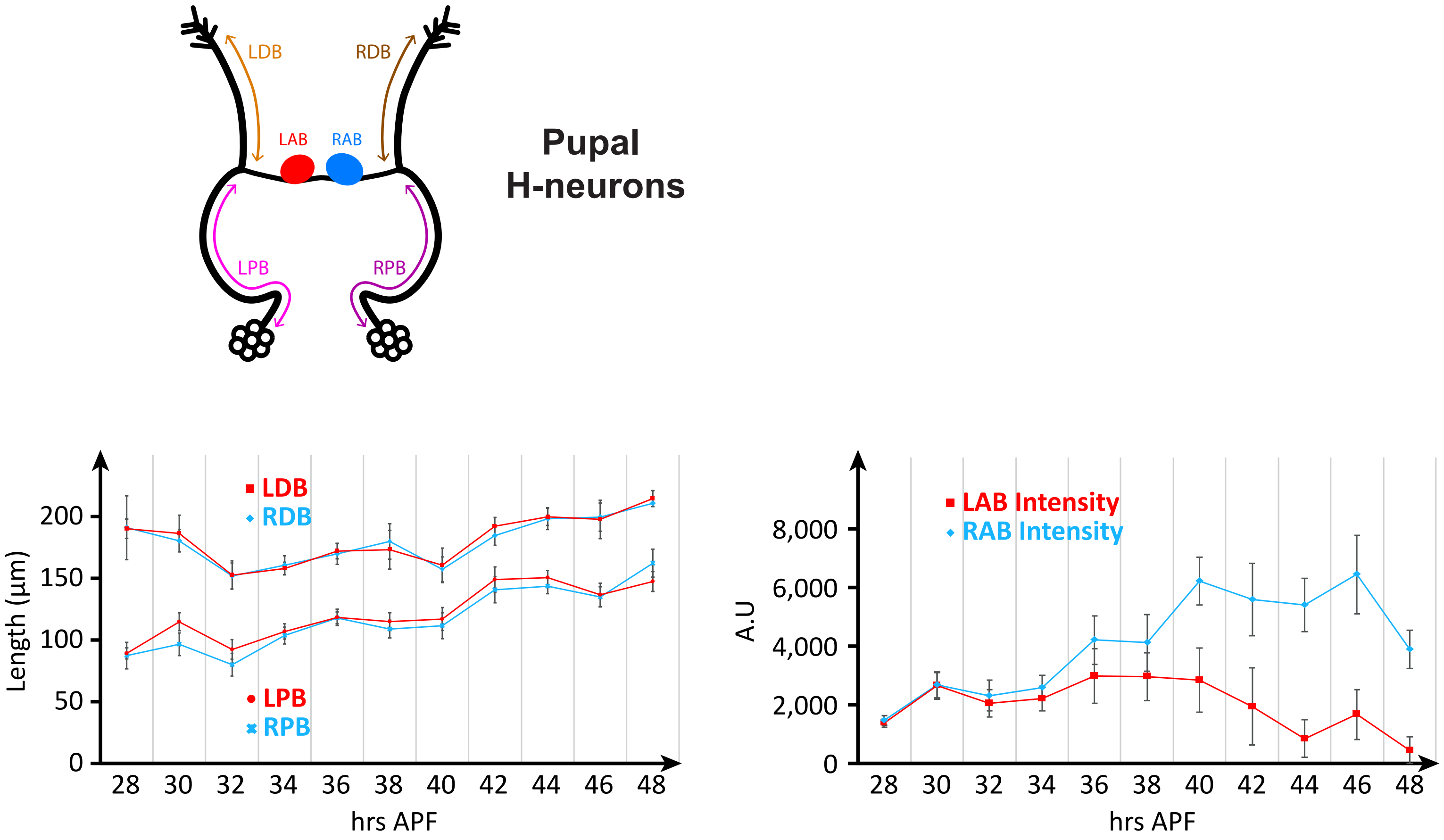

LDB
RDB
LAB
RAB
LPB
RPB
Pupal
H-neurons
LDB
RDB
LPB
RPB
Length (µm)
0
50
100
150
200
28 30 32 34 36 38 40 42 44 46 48
hrs APF
LAB Intensity
RAB Intensity
A.U
0
2,000
4,000
6,000
8,000
28 30 32 34 36 38 40 42 44 46 48
hrs APF

**Supplementary Figure 3: Morphometric analysis of H-neurons in wild-type pupae**

Length of primary and dorsal H neuron branches do not show asymmetry in wild-type flies during pupal stage. Monitoring of 72A10-lexA signal in LAB and RAB show an increasing asymmetry with decrease and increase of the mean intensity in LAB and RAB respectively. n=10 brains for each time point. Values plotted are means ± standard error of the mean (SEM). Source data are provided as a Source Data file.

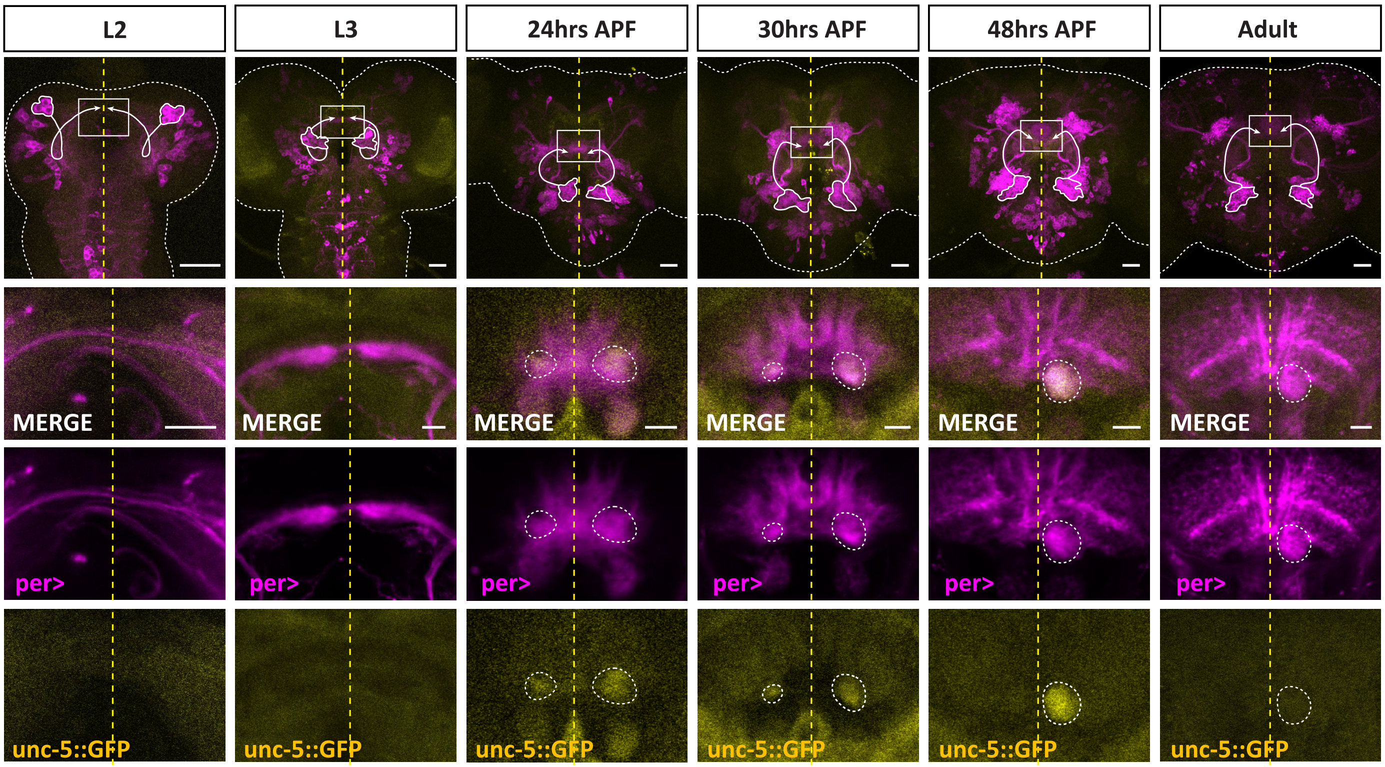

L2
L3
24hrs APF
30hrs APF
48hrs APF
Adult
MERGE
per>
unc-5::GFP

**Supplementary Figure 4: *unc-5::GFP* is expressed in AB during the symmetry-breaking period**

Confocal images of brains expressing mCherry under the control of the *period-Gal4* and an unc-5::GFP fusion protein. ABs express Unc-5 during the “symmetry breaking” period, first symmetrically (in both ABs) then resolving into an asymmetric pattern following the transition of H-neurons in ASYM flies. Expression is not detected at larval stages. Dotted yellow lines indicate brain midline. Dotted white lines indicate brains outline (top panels) and AB positions (bottom panels). Solid white line and arrow indicate LALv1A lineage cell bodies and tracts (upper panels). White squares represent the enlarged area shown in bottom panels. Scale bars represent 30µm for top panels and 10µm for enlarged views in bottom panels. Abbreviations: L2, second instar larvae; L3, third instar larvae; Hrs APF, hours after puparium formation.

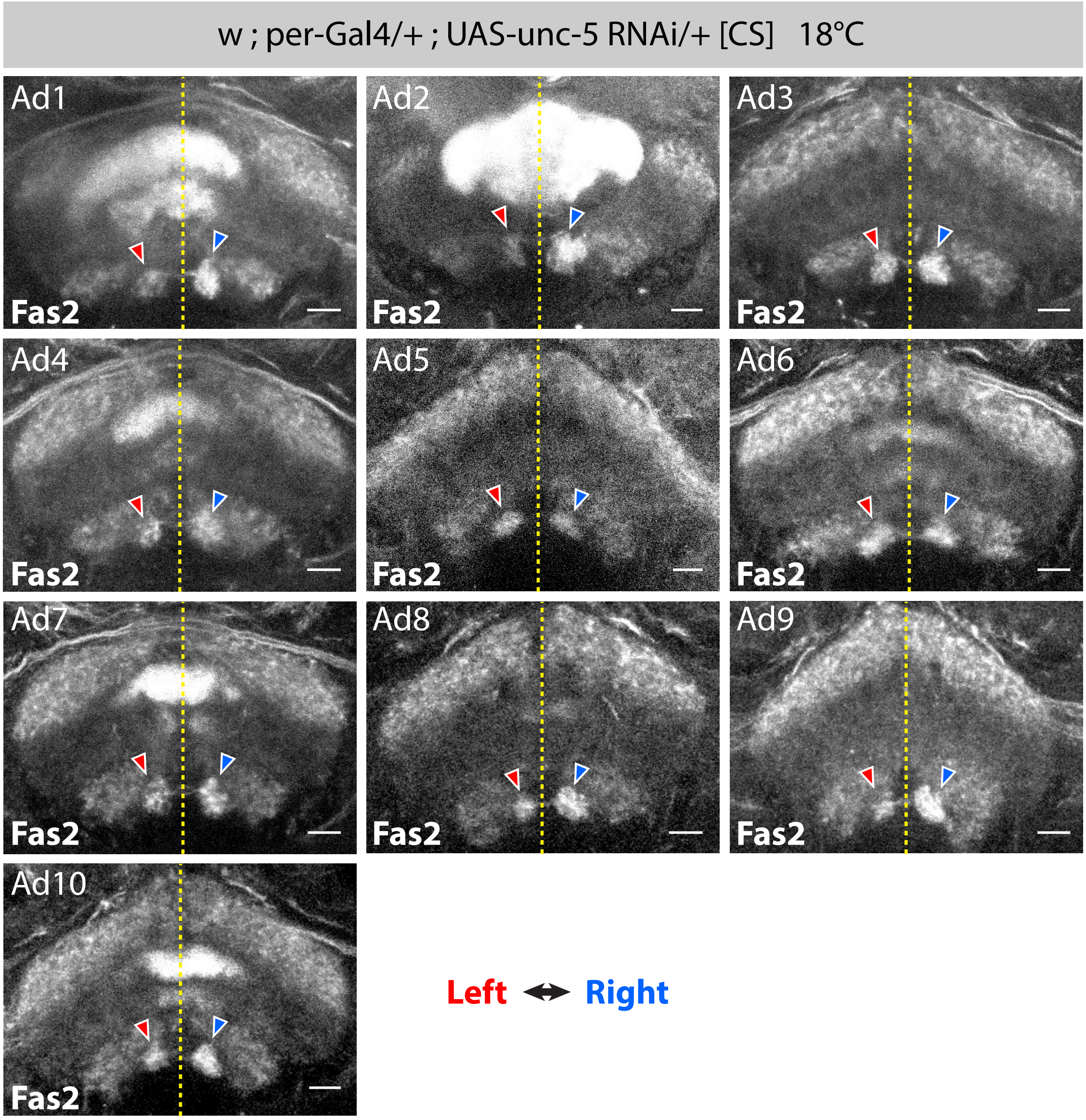
w ; per-Gal4/+ ; UAS-unc-5 RNAi/+ [CS] 18°C
Ad1
Fas2
Ad2
Fas2
Ad3
Fas2
Ad4
Fas2
Ad5
Fas2
Ad6
Fas2
Ad7
Fas2
Ad8
Fas2
Ad9
Fas2
Ad10
Fas2
Left
Right

**Supplementary Figure 5: *unc-5* loss-of-function conditions used for memory testing cause a fully penetrant symmetry phenotype**

Confocal images showing Fas2 antibody labelling in left and right AB (red and blue arrowheads respectively) indicating that adult flies raised at 18°C and expressing unc-5 RNAi using the per-Gal4 driver have a fully penetrant symmetry phenotype. Genotype and temperature used are the same as the one used for the memory tests presented in Fig6b. Yellow dashed line represent brain midline. Scale bars, 10µm.

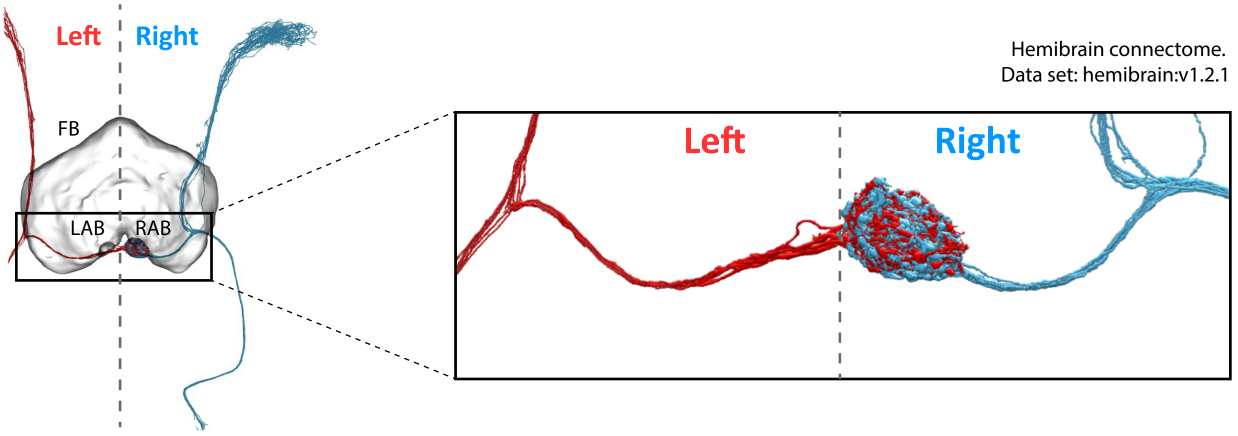


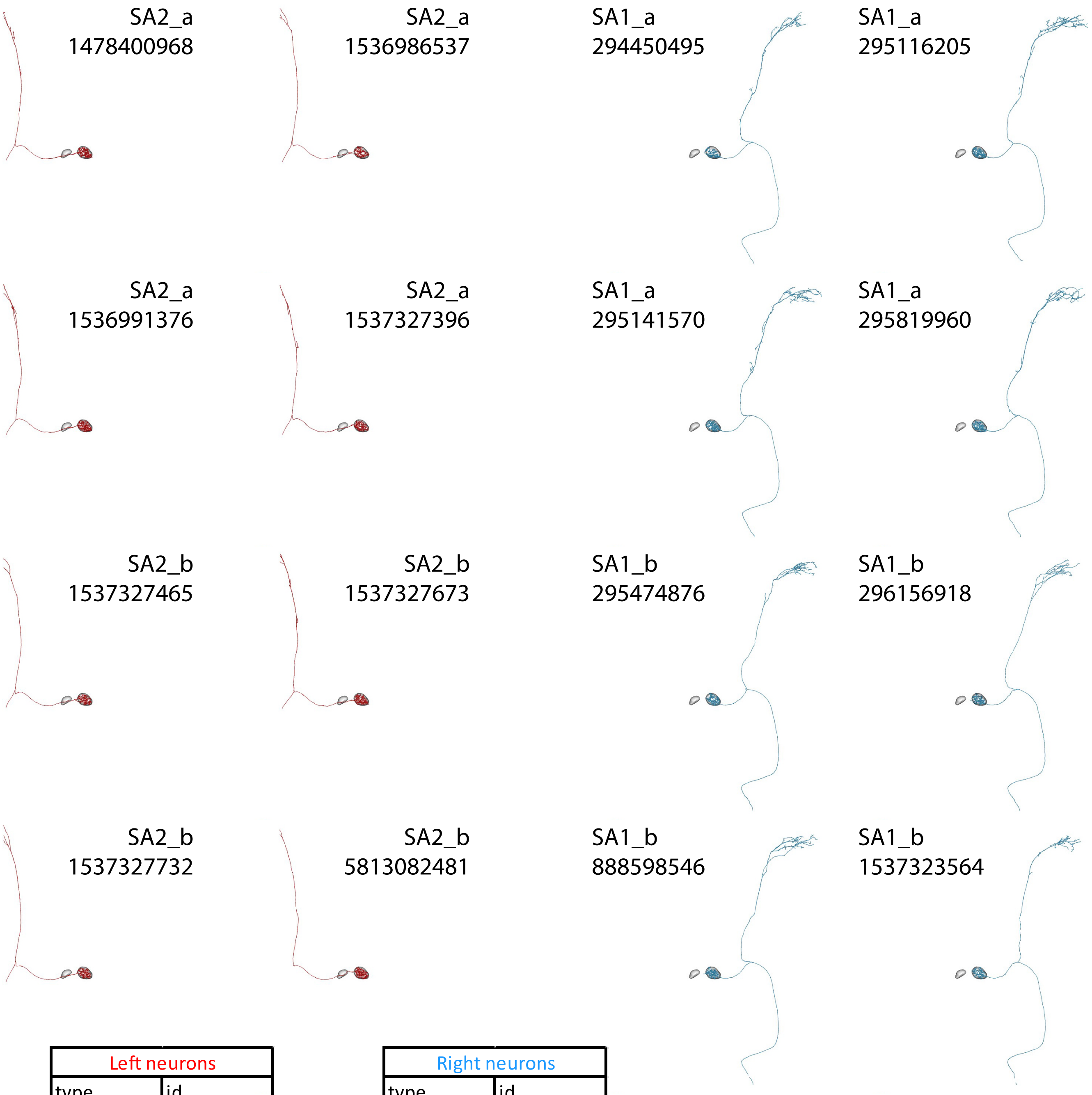


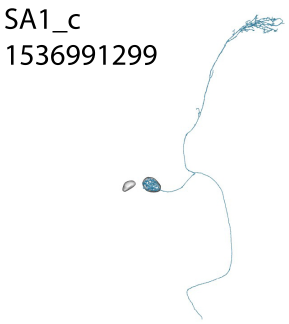


| Left neurons | |
|---|---|
| type | id |
| SA2_a | 1478400968 |
| SA2_a | 1536986537 |
| SA2_a | 1536991376 |
| SA2_a | 1537327396 |
| SA2_b | 1537327465 |
| SA2_b | 1537327673 |
| SA2_b | 1537327732 |
| SA2_b | 5813082481 |

| Right neurons | |
|---|---|
| type | id |
| SA1_a | 294450495 |
| SA1_a | 295116205 |
| SA1_a | 295141570 |
| SA1_a | 295819960 |
| SA1_b | 295474876 |
| SA1_b | 296156918 |
| SA1_b | 888598546 |
| SA1_b | 1537323564 |
| SA1_c | 1536991299 |

**Supplementary Figure 6: H-neuron annotations and morphologies in the hemibrain connectome dataset.**

3D reconstruction and annotation of the 8-left and 9-right neurons matching H-neurons morphology in the connectome dataset from the brain of an adult female individual[76]. Left and right neurons are coloured in red and blue, respectively. Dotted line represents the midline. FB, fan-shaped body; LAB, Left Asymmetrical Body; RAB, Right Asymmetrical Body.

**a**

| | Left | Right |
|---|---|---|
| x-RAB | L-RAB | R-RAB |
| x-LAB | L-LAB | R-LAB |
| x-LRAB | L-LRAB | R-LRAB |

ns 0.166

* 1.91e-2

ns 1

ns 1

ns 1

ns 1

ns 1 | ns 1 | ns 1 | ns 1

| | Left | Right | Left | Right | Left | Right | Left | Right |
|---|---|---|---|---|---|---|---|---|
| | Wildtype ASYM | | Wildtype SYM | | NetB-Delta | | 72A10>unc-5 RNAi | |
| x-RAB | 29 | 19 | 1 | 0 | 1 | 0 | 5 | 6 |
| x-LAB | 0 | 0 | 0 | 0 | 0 | 0 | 0 | 0 |
| x-LRAB | 0 | 0 | 2 | 3 | 10 | 10 | 8 | 7 |

1.11e-2 *

7.14e-8 ***

3.00e-5 ***

1.19e-3 **

2.75e-7 ***

1.19e-3 **

**b**

| | Left + Right |
|---|---|
| x-RAB | L-RAB + R-RAB |
| x-LAB | L-LAB + R-LAB |
| x-LRAB | L-LRAB + R-LRAB |

** 5.67e-3

ns 0.60255

ns 0.60255

| | L+R | L+R | L+R | L+R |
|---|---|---|---|---|
| | Wildtype ASYM | Wildtype SYM | NetB-Delta | 72A10>unc-5 RNAi |
| x-RAB | 48 | 1 | 1 | 11 |
| x-LAB | 0 | 0 | 0 | 0 |
| x-LRAB | 0 | 5 | 20 | 15 |

3.48e-6 ***

9.99e-16 ***

1.16e-8 ***

**Supplementary Figure 7: Statistical analysis of the distinct categories making single H-neurons in wild type and NetB pathway loss-of-function conditions.**

**a,b**. Occurrence of single neuron categories as described in Fig. 1 j-m and Fig. 4 a,b. with counting for left and right neurons separated (**a**) or summed (**b**). Brackets indicate compared conditions and their computed p-Values with two-tailed Fisher's test and Benjamini & Hochberg correction for multiple comparisons. ns, non-significant; *, <0.05 ; **, <0.01 ; ***, < 0.001.
