# Supplementary material for: Asymmetric activity of NetrinB controls laterality of the Drosophila brain
Source: Nat Commun. 2023 Feb 24;14:1052. doi: 10.1038/s41467-023-36644-4 (PMC9958012; doi:10.1038/s41467-023-36644-4)
Supplement: Supplementary file 3 — Description of Additional Supplementary Files [file 41467_2023_36644_MOESM3_ESM.pdf]

Description of Additional Supplementary Files

Supplementary Data 1: Complete scoring table for the RNAi screen

Virgin Females from tester line with genotype "UAS-Dicer2; 72A10-LexA, 13xLex0- 6xmCherry::HA/CyO ; 72A10-Gal4/TM6b" are crossed with males of all "UAS-RNAi" genotypes listed in the table. Crosses are done at 29°C and at least 20 adults (half males half females) from F1 dissected and scored according to H-neurons projections. Statistical analysis is conducted on the "Simple scoring" contingency table with Pearson's Chi-squared test and Benjamini & Yekutieli multiple comparisons correction. "Gene role" in the table is defined as their annotation in [38] and [39] : ML=midline crossing; MA = motor axon guidance; B = both.

Supplementary Data 2: List of genetic crosses and genotypes analyzed.

List of all genetic crosses and genotypes analyzed in the present study.

Supplementary Data 3: List of statistical tests and p-Values

List of all statistical tests and p-values for all manuscript's figures and tables.
