# Supplementary material for: Asymmetric activity of NetrinB controls laterality of the Drosophila brain
Source: Nat Commun. 2023 Feb 24;14:1052. doi: 10.1038/s41467-023-36644-4 (PMC9958012; doi:10.1038/s41467-023-36644-4)
Supplement: Supplementary file 6 — Supplementary Data 3 [file 41467_2023_36644_MOESM6_ESM.docx]

**Supplementary Data 3: List of statistical tests and p-Values**

**Figure 1a**

Done with GraphPad Prism v9.3.1

AB Projection volumes :

- Left vs Right : Two-tailed Wilcoxon test. P-values are :

Wt ASYM : <0.0001 (****) n=16/16

wt SYM : 0.0098 (**) n=10/10

Cell numbers :

- Left vs Right for each conditions : Two-tailed Wilcoxon test. P-values are :

wt ASYM : >0.9999 (ns) n=14/14

wt SYM : 0.6719 (ns) n=9/9

**Figure 3a, b**

Done with R v4.1.1 (chisq.test() and p.adjust())

Pearson’s Chi-squared test with Benjamini & Yekutieli multiple comparisons correction. P-values are :

UAS-Dicer2 ; 72A10>**Stinger** n=182 VS :

UAS-Dicer2 ; 72A10>**unc-5** RNAi #33756 : 5,80E-09 (***) n= 20

UAS-Dicer2 ; 72A10>**Nrx-IV** RNAi #38192 : 4,32E-05 (***) n=21

UAS-Dicer2 ; 72A10>**beat-Ic** RNAi #64528 : 8,24E-05 (***) n= 20

UAS-Dicer2 ; 72A10>**RhoGEF64C** RNAi #77431 : 5,84E-04 (***) n=20

UAS-Dicer2 ; 72A10>**Src64B** RNAi #51772 : 1,34E-03 (**) n=21

UAS-Dicer2 ; 72A10>**daw** RNAi #50911 : 2,66E-03 (**) n= 20

UAS-Dicer2 ; 72A10>**Pak** RNAi #62201 : 2,66E-03 (**) n=20

UAS-Dicer2 ; 72A10>**otk** RNAi #67966 : 6,66E-03 (**) n=30

UAS-Dicer2 ; 72A10>**fra** RNAi #40826 : 4,35E-02 (*) n=24

UAS-Dicer2 ; 72A10>**mud** RNAi #35044 : 4,35E-02 (*) n=24

**Figure 3e**

Done with R v4.1.1 (chisq.test() and p.adjust())

Pearson’s Chi-squared test with Benjamini & Yekutieli multiple comparisons correction. P-values are :

wildtype n=20 VS

NetBΔ : 3,31E-08 (***) n=20

unc-5 MI05371 : 3,31E-08 (***) n=20

NetAΔ : 1,00E+00 (ns) n=20

UAS-Dicer2 ; Elav>Stinger n=20 VS

UAS-Dicer2 ; Elav>NetB RNAi #25861 : 3,01E-08 (***) n=20

UAS-Dicer2 ; Elav>unc-5 RNAi #33756 : 3,01E-08 (***) n=20

UAS-Dicer2 ; Elav>fra RNAi #31664 : 1,48E-03 (**) n=20

UAS-Dicer2 ; Elav>NetA RNA i#330207 : 1,00E+00 (ns) n=20

UAS-Dicer2 ; gcm>Stinger n=20 VS

UAS-Dicer2 ; gcm>NetB RNA i#25861 : 0,9754167 (ns) n=20

UAS-Dicer2 ; gcm>unc-5 RNA i#33756 : 0,9754167 (ns) n=20

UAS-Dicer2 ; gcm>fra RNA i#40826 : 0,9754167 (ns) n=20

UAS-Dicer2 ; gcm>NetA RNAi #330207 : 0,9754167 (ns) n=20

UAS-Dicer2 ; per>Stinger n=21 VS

UAS-Dicer2 ; per>NetB RNAi #25861 : 3,01E-08 (***) n=20

UAS-Dicer2 ; per>unc-5 RNAi #33756 : 3,01E-08 (***) n=20

UAS-Dicer2 ; per>fra RNAi #40826 : 3,94E-03 (*) n=20

UAS-Dicer2 ; per>NetA RNAi #330207 : 1,00E+00 (ns) n=20

UAS-Dicer2 ; 72A10∩VT017124>Stinger n=20 VS

UAS-Dicer2 ; 72A10∩VT017124>NetB RNAi #25861 : 9,35E-01 (ns) n=20

UAS-Dicer2 ; 72A10∩VT017124>unc-5 RNA i#33756 : 3,70E-04 (***) n=20

UAS-Dicer2 ; 72A10∩VT017124>fra RNAi #40826 : 1,36E-04 (***) n=20

UAS-Dicer2 ; 72A10∩VT017124>NetA RNAi #31288 : 9,35E-01 (ns) n=20

wildtype n=20 VS

UAS-Dicer2 ; Elav>Stinger : 1 (ns) n=20

UAS-Dicer2 ; gcm>Stinger : 1 (ns) n=20

UAS-Dicer2 ; per>Stinger : 1 (ns) n=21

UAS-Dicer2 ; 72A10∩VT017124>Stinger : 1 (ns) n=20

**Figure 5b**

Done with R v4.1.1 (fisher.test() and p.adjust())

Two-tailed Fisher’s test with Benjamini & Hochberg multiple comparisons correction. P-values are :

unc-5 single clones

unc-5 RNAi negative control n=110 VS

unc-5 RNAi positive control : 2,83E-32 (***) n=58

unc-5 RNAi single clone Left-ALv1 : 1,00E+00 (ns) n=2

unc-5 RNAi single clone Right-ALv1 : 1,00E+00 (ns) n=2

unc-5 RNAi single clone Left-Ventral1 : 1,00E+00 (ns) n=6

unc-5 RNAi single clone Right-Ventral1 : 1,00E+00 (ns) n=9

unc-5 RNAi single clone Left-SLPpl1 : 1,00E+00 (ns) n=1

unc-5 RNAi single clone Right-SLPpl1 : 9,98E-01 (ns) n=4

unc-5 RNAi single clone Left-EBa1 : 1,00E+00 (ns) n=9

unc-5 RNAi single clone Right-EBa1 : 8,86E-01 (ns) n=7

unc-5 RNAi single clone Left-BAlp2 : 1,00E+00 (ns) n=5

unc-5 RNAi single clone Right-BAlp2 : 9,28E-01 (ns) n=3

unc-5 RNAi single clone Left-DPLm2 : 1,00E+00 (ns) n=6

unc-5 RNAi single clone Right-DPLm2 : 1,00E+00 (ns) n=3

unc-5 RNAi single clone Left-LALv1A : 7,21E-03 (**) n=5

unc-5 RNAi single clone Right-LALv1A : 1,55E-06 (***) n=8

unc-5 RNAi single clone Left-LG-N : 1,00E+00 (ns) n=4

unc-5 RNAi single clone Right-LG-N : 9,28E-01 (ns) n=3

unc-5 RNAi single clone Left/Right-Ventral2 : 1,00E+00 (ns) n=7

unc-5 single+multiple clones

unc-5 RNAi negative control n=110 VS :

unc-5 RNAi positive control : 2,83E-32 (***) n=58

unc-5 RNAi single+multiple clone Left-ALv1 : 1,00E+00 (ns) n=6

unc-5 RNAi single+multiple clone Right-ALv1 : 1,00E+00 (ns) n=6

unc-5 RNAi single+multiple clone Left-Ventral1 : 1,00E+00 (ns) n=14

unc-5 RNAi single+multiple clone Right-Ventral1 : 1,00E+00 (ns) n=22

unc-5 RNAi single+multiple clone Left-SLPpl1 : 1,00E+00 (ns) n=8

unc-5 RNAi single+multiple clone Right-SLPpl1 : 1,00E+00 (ns) n=8

unc-5 RNAi single+multiple clone Left-EBa1 : 1,00E+00 (ns) n=26

unc-5 RNAi single+multiple clone Right-EBa1 : 5,48E-01 (ns) n=16

unc-5 RNAi single+multiple clone Left-BAlp2 : 1,00E+00 (ns) n=14

unc-5 RNAi single+multiple clone Right-BAlp2 : 1,00E+00 (ns) n=8

unc-5 RNAi single+multiple clone Left-DPLm2 : 1,00E+00 (ns) n=15

unc-5 RNAi single+multiple clone Right-DPLm2 : 1,00E+00 (ns) n=13

unc-5 RNAi single+multiple clone Left-LALv1A : 1,32E-08 (***) n=13

unc-5 RNAi single+multiple clone Right-LALv1A : 4,93E-14 (***) n=19

unc-5 RNAi single+multiple clone Left-LG-N : 1,00E+00 (ns) n=9

unc-5 RNAi single+multiple clone Right-LG-N : 7,09E-01 (ns) n=7

unc-5 RNAi single+multiple clone Left/Right-Ventral2 : 1,00E+00 (ns) n=19

**Figure 5c**

Done with R v4.1.1 (fisher.test() and p.adjust())

Two-tailed Fisher’s test with Benjamini & Hochberg multiple comparisons correction. P-values are :

NetB single clones

NetB RNAi negative control n=307 VS :

NetB RNAi positive control : 9,95E-38 (***) n=84

NetB RNAi single clone Left-ALv1 : 1,00E+00 (ns) n=2

NetB RNAi single clone Right-ALv1 : 7,79E-01 (ns) n=3

NetB RNAi single clone Left-Ventral1 : 8,90E-01 (ns) n=8

NetB RNAi single clone Right-Ventral1 : 8,90E-01 (ns) n=11

NetB RNAi single clone Left-SLPpl1 : 1,00E+00 (ns) n=5

NetB RNAi single clone Right-SLPpl1 : 1,00E+00 (ns) n=3

NetB RNAi single clone Left-EBa1 : 1,00E+00 (ns) n=13

NetB RNAi single clone Right-EBa1 : 8,90E-01 (ns) n=10

NetB RNAi single clone Left-BAlp2 : 1,00E+00 (ns) n=4

NetB RNAi single clone Right-BAlp2 : 1,00E+00 (ns) n=5

NetB RNAi single clone Left-DPLm2 : 1,00E+00 (ns) n=5

NetB RNAi single clone Right-DPLm2 : 1,00E+00 (ns) n=3

NetB RNAi single clone Left-LALv1A : 8,90E-01 (ns) n=7

NetB RNAi single clone Right-LALv1A : 9,43E-03 (**) n=5

NetB RNAi single clone Left-LG-N : 1,00E+00 (ns) n=1

NetB RNAi single clone Right-LG-N : 1,00E+00 (ns) n=9

NetB RNAi single clone Left/Right-Ventral2 : 8,90E-01 (ns) n=8

NetB single+multiple clones

NetB RNAi negative control n=307 VS :

NetB RNAi positive control : 9,95E-38 (***) n=84

NetB RNAi single+multiple clone Left-ALv1 : 1,00E+00 (ns) n=12

NetB RNAi single+multiple clone Right-ALv1 : 4,13E-01 (ns) n=7

NetB RNAi single+multiple clone Left-Ventral1 : 6,02E-02 (ns) n=18

NetB RNAi single+multiple clone Right-Ventral1 : 4,06E-01 (ns) n=21

NetB RNAi single+multiple clone Left-SLPpl1 : 7,16E-01 (ns) n=18

NetB RNAi single+multiple clone Right-SLPpl1 : 1,00E+00 (ns) n=12

NetB RNAi single+multiple clone Left-EBa1 : 3,73E-01 (ns) n=29

NetB RNAi single+multiple clone Right-EBa1 : 5,23E-01 (ns) n=29

NetB RNAi single+multiple clone Left-BAlp2 : 3,73E-01 (ns) n=16

NetB RNAi single+multiple clone Right-BAlp2 : 1,00E+00 (ns) n=10

NetB RNAi single+multiple clone Left-DPLm2 : 1,00E+00 (ns) n=10

NetB RNAi single+multiple clone Right-DPLm2 : 3,56E-01 (ns) n=13

NetB RNAi single+multiple clone Left-LALv1A : 3,97E-01 (ns) n=18

NetB RNAi single+multiple clone Right-LALv1A : 6,86E-05 (***) n=11

NetB RNAi single+multiple clone Left-LG-N : 4,06E-01 (ns) n=6

NetB RNAi single+multiple clone Right-LG-N : 2,96E-01 (ns) n=21

NetB RNAi single+multiple clone Left/Right-Ventral2 : 2,96E-01 (ns) n=22

**Figure 6b**

Done with GraphPad Prism v9.4.1

1-way ANOVA followed by Tukey’s multiple comparisons:

5x spaced, 24 hrs memory:

per-GAL4/+ n=14; +/UAS-unc5 RNAi n=14; per-GAL4>UAS-unc5 RNAi n=14.

ANOVA F_2,41_ = 5.47; *p* = 0.0081 (**). Pairwise: per-GAL4/+ *vs* per-GAL4>UAS-unc5 RNAi: *p* = 0.0131 (*); +/UAS-unc5 RNAi *vs* per-GAL4>UAS-unc5 RNAi: *p* = 0.0257 (*); per-GAL4/+ *vs* +/UAS-unc5 RNAi: *p* = 0.9609 (ns)

5x massed, 24 hrs memory:

per-GAL4/+ n=18; +/UAS-unc5 RNAi n=18; per-GAL4>UAS-unc5 RNAi n=18.

ANOVA F_2,53_ = 1.63; *p* = 0.2055 (ns). Pairwise: per-GAL4/+ *vs* per-GAL4>UAS-unc5 RNAi: *p* = 0.1776 (ns); +/UAS-unc5 RNAi *vs* per-GAL4>UAS-unc5 RNAi: *p* = 0.6531 (ns); per-GAL4/+ *vs* +/UAS-unc5 RNAi: *p* = 0.6283 (ns)

1x, 3 hr memory, no cold shock:

per-GAL4/+ n=10; +/UAS-unc5 RNAi n=10; per-GAL4>UAS-unc5 RNAi n=10.

ANOVA F_2,29_ = 0.92; *p* = 0.4123 (ns). Pairwise: per-GAL4/+ *vs* per-GAL4>UAS-unc5 RNAi: *p* = 0.5129 (ns); +/UAS-unc5 RNAi *vs* per-GAL4>UAS-unc5 RNAi: *p* = 0.4512 (ns); per-GAL4/+ *vs* +/UAS-unc5 RNAi: *p* = 0.9939 (ns)

1x, 3 hr memory, with cold shock:

per-GAL4/+ n=9; +/UAS-unc5 RNAi n=9; per-GAL4>UAS-unc5 RNAi n=9.

ANOVA F_2,26_ = 1.21; *p* = 0.3165 (ns). Pairwise: per-GAL4/+ *vs* per-GAL4>UAS-unc5 RNAi: *p* = 0.9968 (ns); +/UAS-unc5 RNAi *vs* per-GAL4>UAS-unc5 RNAi: *p* = 0.3656 (ns); per-GAL4/+ *vs* +/UAS-unc5 RNAi: *p* = 0.4058 (ns)

**Figure 6c**

Done with GraphPad Prism v9.41

1-way ANOVA followed by Tukey’s multiple comparisons:

3x spaced, 24 hrs memory:

per-GAL4 n=40; UAS-unc5 RNAi n=44; per-GAL4>UAS-unc5 RNAi n=56.

ANOVA F_2,139_ = 5.42; *p* = 0.0054 (**). Pairwise: per-GAL4 *vs* per-GAL4>UAS-unc5 RNAi: *p* = 0.0072 (**); UAS-unc5 RNAi *vs* per-GAL4>UAS-unc5 RNAi: *p* = 0.0480 (*); per-GAL4 *vs* UAS-unc5 RNAi: *p* = 0.7571 (ns)

1x, 30m memory:

per-GAL4 n=35; UAS-unc5 RNAi n=33; per-GAL4>UAS-unc5 RNAi n=43

ANOVA F_2,110_ = 1.56; *p* = 0.2151 (ns). Pairwise: per-GAL4 *vs* per-GAL4>UAS-unc5 RNAi: *p* = 0.4866 (ns); UAS-unc5 RNAi *vs* per-GAL4>UAS-unc5 RNAi: *p* = 0.2060 (ns); per-GAL4 *vs* UAS-unc5 RNAi: *p* = 0.8447 (ns)

**Supp Figure 1a-c**

Done with GraphPad Prism v9.3.1

AB Projection volumes

- Left vs Right : Two-tailed Wilcoxon test. P-values are :

Wt ASYM : <0.0001 (****) n=16/16

wt SYM : 0.0098 (**) n=10/10

NetB Delta : <0.0001 (****) n=17/17

72A10>unc-5 RNAi : 0.0002 (***) n=13/13

- Left+Right total : Krustal-Wallis test with Dunn’s multiple comparisons correction. P-values are :

wt ASYM vs wt SYM: >0.9176 (ns) n=26/10

wt ASYM vs NetB Delta : <0.0001 (****) n=26/17

wt ASYM vs 72A10>unc-5 RNAi : >0.9999 (ns) n=26/13

- Left/Right ratios : Two-tailed Mann-Whitney test

wt SYM vs NetB Delta : 0.9019 (ns) n=10/17

wt SYM vs 72A10>unc-5 RNAi : 0.0666 (ns) n=10/13

NetB Delta vs 72A10>unc-5 RNAi : 0.0249 (*) n=17/13

**Supp Figure 1b**

Done with GraphPad Prism v9.3.1

Cell numbers :

- Left vs Right for each conditions : Two-tailed Wilcoxon test. P-values are :

wt ASYM : >0.9999 (ns) n=14/14

wt SYM : 0.6719 (ns) n=9/9

NetB Delta : >0.9999 (ns) n=16/16

72A10>unc-5 RNAi : 0.4014 (ns) n=12/12

- Left vs Left and Right vs Right : Krustal-Wallis test with Dunn’s multiple comparisons correction. P-values are :

L wt ASYM vs L wt SYM: >0.9999 (ns) n=14/9

L wt ASYM vs L NetB Delta : >0.9999 (ns) n=14/16

L wt ASYM vs L 72A10>unc-5 RNAi : >0.9999 (ns) n=14/12

R wt ASYM vs R wt SYM: >0.9999 (ns) n=14/9

R wt ASYM vs R NetB Delta : >0.9999 (ns) n=14/16

R wt ASYM vs R 72A10>unc-5 RNAi : 0.8917 (ns) n=14/12

**Supp Figure 1c**

Done with GraphPad Prism v9.3.1

Dorsal branch lengths :

- Left vs Right : Two-tailed Wilcoxon test. P-values are :

Wt ASYM : 0,1722 (ns) n=15/15

wt SYM : 0.9688 (ns) n =8/8

NetB Delta : 0.6788 (ns) n =17/17

72A10>unc-5 RNAi : 0.3921 (ns) n =13/13

Medial branch lengths :

- Left vs Right : Two-tailed Wilcoxon test. P-values are :

Wt ASYM : <0.0001 (****) n=15/15

wt SYM : 0.3125 (ns) n =8/8

NetB Delta : 0.1558 (ns) n =17/17

72A10>unc-5 RNAi : 0.4854 (ns) n =13/13

Primary branch lengths :

- Left vs Right : Two-tailed Wilcoxon test. P-values are :

Wt ASYM : 0.4974 (ns) n=15/15

wt SYM : 0.2109 (ns) n =8/8

NetB Delta : 0.4799 (ns) n =17/17

72A10>unc-5 RNAi : 0.8394 (ns) n =13/13

**Supp Figure 2a**

Done with R v4.1.1 (chisq.test() and p.adjust())

Pearson’s Chi-squared test with Benjamini & Yekutieli multiple comparisons correction. P-values are :

Control n=20 VS

stH99/+ : 1,00E+00 (ns) n=20

per>P35 : 1,00E+00 (ns) n=20

**Supp Figure 2b**

Done with R v4.1.1 (chisq.test())

Pearson’s Chi-squared test. P-values are :

Control n=20 VS

Myo1D[K2] : 1,00E+00 (ns) n=36

**Supp Figure 2c**

Done with R v4.1.1 (chisq.test() and p.adjust())

Pearson’s Chi-squared test with Benjamini & Yekutieli multiple comparisons correction. P-values are :

UAS-Dicer2 ; gcm>Stinger n=20 VS

UAS-Dicer2 ; gcm>**Nrx-IV** RNAi #38192 : 1,00E+00 (ns) n=21

UAS-Dicer2 ; gcm>**Src64B** RNAi #51772 : 1,00E+00 (ns) n=20

UAS-Dicer2 ; gcm>**mud** RNAi #35044 : 1,00E+00 (ns) n=20

UAS-Dicer2 ; gcm>**otk** RNAi #67966 : 1,00E+00 (ns) n=20

UAS-Dicer2 ; gcm>**daw** RNAi #50911 : NA (ns) n= 20

UAS-Dicer2 ; gcm>**beat-Ic** RNAi #64528 : 1,00E+00 (ns) n= 20

UAS-Dicer2 ; gcm>**RhoGEF64C** RNAi #77431 : NA (ns) n=20

UAS-Dicer2 ; gcm>**Pak** RNAi #62201 : 1,00E+00 (ns) n=20

UAS-Dicer2 ; gcm>**fra** RNAi #40826 : 1,00E+00 (ns) n=20

UAS-Dicer2 ; gcm>**unc-5** RNAi #33756 : 1,00E+00 (ns) n= 20

**Supp Figure 2d**

Done with R v4.1.1 (chisq.test() and p.adjust())

Pearson’s Chi-squared test with Benjamini & Yekutieli multiple comparisons correction. P-values are :

Control n=20 VS

NetA-Delta : 1,00E+00 (ns) n=20

NetB-Delta : 1,09E-07 (***) n=20

NetB-Delta/+ : 2,17E-01 (ns) n=20

NetA-Delta, NetB-myc : 1,09E-07 (***) n=20

NetA-Delta, NetB-myc/+ : 1,00E+00 (ns) n=20

NetA-Delta, NetB-myc/NetB-Delta : 1,09E-07 (***) n=20

NetA-Delta, NetB-TM : 1,09E-07 (***) n=20

NetA-Delta, NetB-TM/+ : 2,44E-06 (***) n=20

NetB-TJ>/+ ; >RFP/+ : 9,18E-06 (***) n=20

NetB::GFP MI10467 : 1,09E-07 (***) n=20

NetB::GFP MI10467/+ : 1,01E-05 (***) n=20

NetB::GFP BA00253 : 5,15E-07 (***) n=20

NetB::GFP BA00253/+ : 7,66E-01 (ns) n=20

NetB::Venus CPTI00168 : 1,54E-07 (***) n=19

NetB::Venus CPTI00168/+ : 1,09E-07 (***) n=20

unc-5::GFP MI05371-GFSTF.1/+ : 1,00E+00 (ns) n=20

NetA-Delta n=20 VS

NetA-Delta, NetB-myc : 5,15E-09 (***) n=20

NetA-Delta, NetB-myc/+ : NA (ns) n=20

NetA-Delta, NetB-TM : 5,15E-09 (***) n=20

NetA-Delta, NetB-TM/+ : 1,20E-07 (***) n=20

NetA-Delta, NetB-myc/NetB-Delta n=20 VS

NetA-Delta, NetB-myc/+ : 5,62E-09 (***) n=20

NetB-Delta/+ : 7,65E-05 (***) n=20

**Supp Figure 7a**

Done with R v4.1.1 (fisher.test() and p.adjust())

Two-tailed Fisher’s test with Benjamini & Hochberg multiple comparisons correction. P-values are :

Left wild type ASYM n=29 VS

Left wild type SYM : 1,11E-02 (*) n=3

Left NetB-Delta : 7,14E-08 (***) n=11

Left 72A10>unc-5 RNAi : 3,00E-05 (***) n=13

Right wild type ASYM n=19 VS

Right wild type SYM : 1,19E-03 (**) n=3

Right NetB-Delta : 2,75E-07 (***) n=10

Right 72A10>unc-5 RNAi : 1,19E-03 (**) n=13

Left wild type SYM n=3 VS

Left NetB-Delta : 1 (ns) n=11

Left 72A10>unc-5 RNAi : 1 (ns) n=13

Right wild type SYM n=3 VS

Right NetB-Delta : 1 (ns) n=10

Right 72A10>unc-5 RNAi : 1 (ns) n=13

Done with R v4.1.1 (fisher.test())

Two-tailed Fisher’s test. P-values are :

Left NetB-Delta n=11 VS

Left 72A10>unc-5 RNAi : 0,166 (ns) n=13

Right NetB-Delta n=10 VS

Right 72A10>unc-5 RNAi : 1,91E-02 (*) n=13

Left wild type ASYM n=29 VS

Right wild type ASYM : 1 (ns) n=19

Left wild type SYM n=3 VS

Right wild type SYM : 1 (ns) n=3

Left NetB-Delta n=11 VS

Right NetB-Delta : 1 (ns) n=10

Left 72A10>unc-5 RNAi n=13 VS

Right 72A10>unc-5 RNAi : 1 (ns) n=13

**Supp Figure 7b**

Done with R v4.1.1 (fisher.test() and p.adjust())

Two-tailed Fisher’s test with Benjamini & Hochberg multiple comparisons correction. P-values are :

Wild type ASYM n=48 VS

Wild type SYM : 3,48E-06 (***) n=6

NetB-Delta : 9,99E-16 (***) n=21

72A10>unc-5 RNAi : 1,16E-08 (***) n=26

Wild type SYM n=6 VS

NetB-Delta : 0,60255 (ns) n=21

72A10>unc-5 RNAi : 0,60255 (ns) n=26

Done with R v4.1.1 (fisher.test())

Two-tailed Fisher’s test. P-values are :

NetB-Delta n=21 VS

72A10>unc-5 RNAi : 5,67E-03 (**) n=26
