# Supplementary material for: Asymmetric activity of NetrinB controls laterality of the Drosophila brain
Source: Nat Commun. 2023 Feb 24;14:1052. doi: 10.1038/s41467-023-36644-4 (PMC9958012; doi:10.1038/s41467-023-36644-4)
Supplement: Supplementary file 7 — Reporting Summary [file 41467_2023_36644_MOESM7_ESM.pdf]

nature portfolio

Corresponding author(s): Stéphane Noselli, François Lapraz

Last updated by author(s): Dec 15, 2022

Reporting Summary

Nature Portfolio wishes to improve the reproducibility of the work that we publish. This form provides structure for consistency and transparency in reporting. For further information on Nature Portfolio policies, see our Editorial Policies and the Editorial Policy Checklist.

Statistics

For all statistical analyses, confirm that the following items are present in the figure legend, table legend, main text, or Methods section.

n/a	Confirmed	
☐	☒	The exact sample size (n) for each experimental group/condition, given as a discrete number and unit of measurement
☐	☒	A statement on whether measurements were taken from distinct samples or whether the same sample was measured repeatedly
☐	☒	The statistical test(s) used AND whether they are one- or two-sided<br>*Only common tests should be described solely by name; describe more complex techniques in the Methods section.*
☒	☐	A description of all covariates tested
☐	☒	A description of any assumptions or corrections, such as tests of normality and adjustment for multiple comparisons
☐	☒	A full description of the statistical parameters including central tendency (e.g. means) or other basic estimates (e.g. regression coefficient) AND variation (e.g. standard deviation) or associated estimates of uncertainty (e.g. confidence intervals)
☐	☒	For null hypothesis testing, the test statistic (e.g. F, t, r) with confidence intervals, effect sizes, degrees of freedom and P value noted<br>*Give P values as exact values whenever suitable.*
☒	☐	For Bayesian analysis, information on the choice of priors and Markov chain Monte Carlo settings
☒	☐	For hierarchical and complex designs, identification of the appropriate level for tests and full reporting of outcomes
☒	☐	Estimates of effect sizes (e.g. Cohen's d, Pearson's r), indicating how they were calculated

Our web collection on statistics for biologists contains articles on many of the points above.

Software and code

Policy information about availability of computer code

Data collection	Zen (Black edition) was used for all confocal acquisitions
Data analysis	Fiji v1.52p was used for confocal image visualisation. Fiji's CMTK registration plugin was used to align clone labellings to the Tefor reference brain / Microsoft Excel 2016 / GraphPad Prism (v9.3.1 and v9.4.1) and R v4.1.1 were used for statistical analysis / Imaris v9.6.1 (Oxford instrument) was used for morphometric analysis / R v4.1.1 and ggplot2 v3.2.2 package was used to calculate local regression curves for RNAi sensitive period data visualization. Neuprint v1.2.1 connectome dataset with “Find neurons” and “vizualisation/skeleton” tools were used to identify and visualize H-neurons (neuprint.janelia.org).

For manuscripts utilizing custom algorithms or software that are central to the research but not yet described in published literature, software must be made available to editors and reviewers. We strongly encourage code deposition in a community repository (e.g. GitHub). See the Nature Portfolio guidelines for submitting code & software for further information.

Data

Policy information about availability of data

All manuscripts must include a data availability statement. This statement should provide the following information, where applicable:

- Accession codes, unique identifiers, or web links for publicly available datasets
- A description of any restrictions on data availability
- For clinical datasets or third party data, please ensure that the statement adheres to our policy

All the data generated with this work is provided as “Source data file” and “Supplementary tables“

Human research participants

Policy information about studies involving human research participants and Sex and Gender in Research.

Reporting on sex and gender	not applicable
Population characteristics	not applicable
Recruitment	not applicable
Ethics oversight	not applicable

Note that full information on the approval of the study protocol must also be provided in the manuscript.

Field-specific reporting

Please select the one below that is the best fit for your research. If you are not sure, read the appropriate sections before making your selection.

☒ Life sciences ☐ Behavioural & social sciences ☐ Ecological, evolutionary & environmental sciences

For a reference copy of the document with all sections, see nature.com/documents/nr-reporting-summary-flat.pdf

Life sciences study design

All studies must disclose on these points even when the disclosure is negative.

Sample size	Sample target size of n=20 individuals was chosen according to standard used in previously published Drosophila phenotypic caracterization. Statistical power calculated with this sample size is : Chi2 test power = 0.61 / Difference of proportion power = 0.72.<br>(Power calulations done with R v4.1.1 "pwr" package and the pwr.chisq.test (signal level=0.05 ; degree freedom = 1 ; size effect = 0.5) and pwr.2p.test (signal level=0.05 ; size effect = 0.8))
Data exclusions	No data was excluded from our analysis
Replication	Experiments were typically replicated 2 to 3 times. For RNAi screen, clonal analysis and memory testings results were gathered from multiple independant experiments.
Randomization	For each experiment, animals with appropriate genotype were randomly allocated to experimental groups.
Blinding	Investigators were not blind to group allocation during data collection. Group allocation is determined by genotype in the present study which, in Drosophila, manifests by the presence or absence of phenotypic markers that cannot be blinded to the experimentator when dissecting/analysing the animals.

Reporting for specific materials, systems and methods

We require information from authors about some types of materials, experimental systems and methods used in many studies. Here, indicate whether each material, system or method listed is relevant to your study. If you are not sure if a list item applies to your research, read the appropriate section before selecting a response.

Materials & experimental systems

n/a	Involved in the study	
☐	☒	Antibodies
☒	☐	Eukaryotic cell lines
☒	☐	Palaeontology and archaeology
☐	☒	Animals and other organisms
☒	☐	Clinical data
☒	☐	Dual use research of concern

Methods

n/a	Involved in the study	
☒	☐	ChIP-seq
☒	☐	Flow cytometry
☒	☐	MRI-based neuroimaging

Antibodies

Antibodies used

Primary antibodies: Mouse anti-brp (Nc82) (Developmental Studies Hybridoma Bank - Ref nc82 - 1:100), Mouse anti-Fas2 (Developmental Studies Hybridoma Bank - Ref 1D4), Goat anti-GFP (Antibodies-online – Ref ABIN100085 – 1:200), Mouse anti-V5 (Invitrogen – Ref 46-0705 (new Ref R960-25) – 1:300), Rat anti-Flag (Novus – Ref NBP1-06712SS – 1:300), Rabbit anti-Flag (Sigma – Ref F7425 – 1:300).

Secondary antibodies: Donkey anti-mouse Alexa Fluor 647 (Invitrogen - Ref A31571 - 1:500), Donkey anti-mouse Alexa Fluor 488 (Invitrogen - Ref A21202 – 1:500), Donkey anti-rat Alexa Fluor 647 (Invitrogen – Ref A21247 - 1:500), Donkey anti-rabbit Alexa Fluor 647 (Invitrogen - Ref A31573 - 1:500), Donkey anti-goat Alexa Fluor 488 (Invitrogen – Ref A-11055 – 1:500).

Validation

Mouse anti-Brp (Developmental Studies Hybridoma Bank - Ref Nc82) Monoclonal. Has been validated by a large number of study as marker of presynaptic active zones (Wagh et al. 2006 ; DOI: 10.1016/j.neuron.2006.02.008) and is widely used as a standard to label Drosophila neuropils (e.g Arganda-Carreras et al. 2018 ; DOI: 10.3389/fninf.2018.00013).

Mouse anti-Fas2 (Developmental Studies Hybridoma Bank - Ref 1D4) Monoclonal. Has been validated as a marker for asymmetrical body in Pascual et al. 2004 (DOI: 10.1038/427605a).

Goat anti-GFP (Antibodies-online – Ref ABIN100085), Polyclonal. Raised against Aequorea victoria GFP and validated for detection of GFP and its variants (rGFP, eGFP) in Immunofluorescence, ELISA, and Western Blot.

Mouse anti-V5 (Invitrogen – Ref 46-0705 (new Ref R960-25)), Monoclonal. Raised against V5 synthetic peptide (GKPIPNPLLGLDST) and validated for detection of V5-tagged proteins in Western Blot, Immunohistochemistry, Immunocytochemistry, Flow Cytometry, ELISA, Immunoprecipitation, ChIP assay, in situ PLA, Dot blot, Inhibition Assays and RNA Immunoprecipitation.

Rat anti-Flag (Novus – Ref NBP1-06712SS), Monoclonal. Raised against N-terminal DYKDDDDK-tagged extracellular domain of mouse Langerin and validated for detection of Flag-tagged proteins in Western Blot, Immunohistochemistry and Immunocytochemistry.

Rabbit anti-Flag (Sigma – Ref F7425), Polyclonal. Raised against peptide sequence DYKDDDDK and validated for detection of Flag-tagged proteins in dot blot, immunoprecipitation, immunofluorescence and western blot.

Animals and other research organisms

Policy information about studies involving animals; ARRIVE guidelines recommended for reporting animal research, and Sex and Gender in Research

Laboratory animals

Larva, pupa and adult (2 to 5 days) Drosophila melanogaster from various strains were used in this study.

Strains (and their origin) used is this study are:
Stocks obtained from the Bloomington Drosophila Stock Center: 72A10-Gal4 (#48306) ; 72A10-LexA (#54191) ; 72A10-p65AD (#70799) ; Elav-Gal4 (#8765) ; gcm-Gal4 (#35541) ; NetB-Gal4 (#76730) ; per-Gal4 (#7127) ; VT017124-GDBD (#75461) ; 38D01-Gal4 (#49996) ; NetA-Delta, NetB-TM (#66880) ; NetB::GFP BA00253 (#50794) ; NetB::GFP MI10467 (#67644) ; unc-5::GFP MI05371 (#60547) ; NetA-Delta, (#66878) ; NetB-Delta (#66879) ; unc-5 MI05371 (#42316) ; fra[3] (#8813) ; fra[4] (#8743) ; 13xLexAop2-6xEGFP (#52265 and #52266) ; 13xLexAop2-6xmCherryHA (#52271 and #52272) ; 20XUAS-6xEGFP (#52261 and #52262) ; 20XUAS-6xmCherryHA (#52267 and #52268) ; UAS-Stinger (#84278 and #65402) ; FRT19A (#1709) ; hsFlp, FRT19A, tubP-Gal80ts (#5133) ; MCFO1 (#64085) ; MCFO2 (#64086) ; MCFO4 (#64088) ; MCFO5 (#64089) ; Df(3L)99 (#1576) ; tubP-Gal80ts (#7019) ; UAS-Dicer2 (#24646 and #24651) ; UAS-P35 (#5073). UAS Abl RNAi (#28325) ; UAS Ama RNAi (#33416) ; UAS babo RNAi (#40866) ; UAS babo RNAi (#55871) ; UAS beat-Ia RNAi (#64938) ; UAS beat-Ic RNAi (#64528) ; UAS beta-Spec RNAi (#38533) ; UAS Cam RNAi (#34609) ; UAS cno RNAi (#33367) ; UAS comm RNAi (#28381) ; UAS Con RNAi (#28967) ; UAS daw RNAi (#34974) ; UAS daw RNAi (#50911) ; UAS dlp RNAi (#34091) ; UAS dock RNAi (#27728) ; UAS dock RNAi (#37524) ; UAS ena RNAi (#31582) ; UAS ena RNAi (#39034) ; UAS Fas2 RNAi (#28990) ; UAS Fas2 RNAi (#34084) ; UAS fra RNAi (#31664) ; UAS fra RNAi (#40826) ; UAS hh RNAi (#25794) ; UAS kuz RNAi (#66958) ; UAS Lar RNAi (#40938) ; UAS mud RNAi (#28074) ; UAS mud RNAi (#35044) ; UAS NetB RNAi (#25861) ; UAS NetB RNAi (#34698) ; UAS Nrg RNAi (#37496) ; UAS Nrt RNAi (#28742) ; UAS Nrx-IV RNAi (#38192) ; UAS otk RNAi (#67966) ; UAS Pak RNAi (#41714) ; UAS Pak RNAi (#62201) ; UAS PlexA RNAi (#30483) ; UAS Psn RNAi (#27681) ; UAS ptc RNAi (#28795) ; UAS Ptp10D RNAi (#39001) ; UAS Ptp52F RNAi (#64940) ; UAS Ptp69D RNAi (#38370) ; UAS Ptp99A RNAi (#57299) ; UAS put RNAi (#39025) ; UAS RhoGAP93B RNAi (#31167) ; UAS RhoGAPp190 RNAi (#43987) ; UAS RhoGEF64C RNAi (#77431) ; UAS robo1 RNAi (#35768) ; UAS robo1 RNAi (#39027) ; UAS robo2 RNAi (#27317) ; UAS robo2 RNAi (#34589) ; UAS robo3 RNAi (#29398) ; UAS robo3 RNAi (#44539) ; UAS Sdc RNAi (#51723) ; UAS Sema1a RNAi (#34320) ; UAS Sema2a RNAi (#29519) ; UAS sli RNAi (#31467) ;

UAS sli RNAi (#31468) ; UAS Sos RNAi (#31597) ; UAS Sos RNAi (#34833) ; UAS sqh RNAi (#33892) ; UAS Src42A RNAi (#55868) ; UAS Src64B RNAi (#51772) ; UAS Src64B RNAi (#62157) ; UAS stan RNAi (#26022) ; UAS trio RNAi (#27732) ; UAS trio RNAi (#43549) ; UAS unc-5 RNAi (#33756) ; UAS Wnt5 RNAi (#34644).
Stock obtained from the Vienna Drosophila Resource Center: UAS Abl RNAi (#110186) ; UAS Ama RNAi (#22945) ; UAS beat-Ia RNAi (#46815) ; UAS beat-Ic RNAi (#105066) ; UAS beta-Spec RNAi (#42054) ; UAS Cam RNAi (#102004) ; UAS cno RNAi (#7769) ; UAS comm RNAi (#110488) ; UAS Con RNAi (#101187) ; UAS dlp RNAi (#100268) ; UAS drl RNAi (#3048) ; UAS drl RNAi (#27053) ; UAS Dscam1 RNAi (#25623) ; UAS Dscam1 RNAi (#108835) ; UAS hh RNAi (#109454) ; UAS kuz RNAi (#330255) ; UAS Lar RNAi (#107926) ; UAS mmy RNAi (#105829) ; UAS mmy RNAi (#330168) ; UAS NetA RNAi (#108577) ; UAS NetA RNAi (#330207) ; UAS Nrg RNAi (#107991) ; UAS Nrt RNAi (#106080) ; UAS Nrx-IV RNAi (#108128) ; UAS otk RNAi (#30833) ; UAS PlexA RNAi (#107004) ; UAS PlexB RNAi (#27220) ; UAS PlexB RNAi (#46687) ; UAS Psn RNAi (#101379) ; UAS ptc RNAi (#330595) ; UAS Ptp10D RNAi (#8010) ; UAS Ptp52F RNAi (#39175) ; UAS Ptp69D RNAi (#104761) ; UAS Ptp99A RNAi (#103931) ; UAS put RNAi (#107071) ; UAS RhoGAP93B RNAi (#100136) ; UAS RhoGAPp190 RNAi (#28877) ; UAS RhoGEF64C RNAi (#105252) ; UAS Sdc RNAi (#13322) ; UAS Sema1a RNAi (#104505) ; UAS Sema2a RNAi (#15810) ; UAS Sema2b RNAi (#101842) ; UAS Sema2b RNAi (#108030) ; UAS side-VI RNAi (#38809) ; UAS side-VI RNAi (#103456) ; UAS Sidpn RNAi (#3065) ; UAS Sidpn RNAi (#27049) ; UAS sqh RNAi (#109493) ; UAS Src42A RNAi (#26019) ; UAS stan RNAi (#107993) ; UAS tutl RNAi (#23715) ; UAS tutl RNAi (#108746) ; UAS unc-5 RNAi (#8138) ; UAS Wnt5 RNAi (#101621).
Stock obtained from the Kyoto Drosophila Stock Center: NetB::GFP CPTI168 (#115011).
Stock generated in Stéphane Noselli's laboratory: Myo1DK2.
Stock provided by other laboratories: NetA-Delta, NetBmyc (Darren Williams) ; w+ [CS] ; w1118 [CS] (Thomas Préat).

Wild animals	This study did not involve wild animals
Reporting on sex	For all genotypes analysed, half females and males were analysed. Only exceptions are due to genetic constraints for transgenes located on X chromosome (e.g heterozygous conditions for transgene located on X chromosome (only females were analyzed), or positive controls for the period clonal analysis (only males were analyzed). All crosses and genotypes analysed are provided as a supplementary Table. For genotypes were both females and males were analyzed, no difference was observed between genders and n numbers presented in this study represent results from both males and females.
Field-collected samples	This study did not involve field-collected samples
Ethics oversight	No ethical approval was required

Note that full information on the approval of the study protocol must also be provided in the manuscript.
